# *Metapocyrtusdagtum* sp. nov., a new flightless weevil from Davao de Oro, Mindanao Island, Philippines (Coleoptera: Curculionidae: Entiminae: Pachyrhynchini)

**DOI:** 10.3897/BDJ.9.e72561

**Published:** 2021-11-02

**Authors:** Analyn Anzano Cabras, Chrestine Torrejos, Milton Norman Medina

**Affiliations:** 1 Coleoptera Research Center, Institute for Biodiversity and Environment, University of Mindanao, Davao City, Philippines Coleoptera Research Center, Institute for Biodiversity and Environment, University of Mindanao Davao City Philippines

**Keywords:** beetles, biodiversity, new species, southern Mindanao, taxonomy

## Abstract

**Background:**

The genus *Metapocyrtus* Heller, 1912 is the most speciose and taxonomically complex genus in the tribe Pachyrhynchini. It is known to be endemic in the Philippines, with most species having a very narrow range of distribution. There are already more than 230 species of *Metapocyrtus* documented in the Philippines.

**New information:**

*Metapocyrtusdagtum* sp. nov., a new species of the genus *Metapocyrtus* Heller, 1912 from Davao de Oro, Mindanao Island, Philippines, is described with brief notes about its ecology. Its specific epithet is from the Cebuano word “dagtum” which means pitch black referring to the colour of the integument of the species.

## Introduction

Davao de Oro, formerly Compostela Valley, is located in the eastern part of Davao Region, bounded by Agusan del Sur in the north, Davao Oriental in the east and the south, Davao Gulf in the southwest, and Davao del Norte in the west. It covers a total area of 4,666.93 km^2^, of which 3,135.96 km^2^ is classified as forestland. The terrain of the Province consists of flat, rolling, hilly, and mountainous portions, with its highest elevation reaching more than 2000m (i.e. Mabini, Maragusan, New Bataan and Pantukan) and the low elevation areas, at a height below 100m (i.e. Monkayo, Laak and Compostela) (National Economic and Development Authority 2021). Most importantly, the Province is part of the the Eastern Mindanao Biodiversity Corridor (EMBC), one of the three identified key biodiversity hotspot areas in the country, which harbours three out of the nine key biodiversity areas (KBAs) and serves as the remaining stronghold for globally-endangered species in the Philippines (Philippine Eagle Foundation et al. 2008). Davao de Oro proves to be a trove of new species as new species of beetles from the genus *MetapocyrtusHeller 1912* (Curculionidae) (Cabras et al. 2018, Cabras and Medina 2019), *Pachyrhynchus* Germar (Rukmane and Barsevskis 2016), *Odochilus* Harold, 1877 (Scarabaeidae) (Rakovič and Anichtchenko 2021), *Rhyparus* Agassiz, 1846 (Scarabaeidae) (Anichtchenko et al. 2021) and *Thopeutica* Schaum, 1861 (Cicindelidae) (Medina et al. 2019) have been described from the Province within the past few years.

*Metapocyrtus* is the most speciose and taxonomically complex genus in the tribe Pachyrhynchini Schönherr, 1826 (Schultze 1925, Yap and Gapud 2007). It is known to be endemic in the Philippines with most species having a very narrow range of distribution (Bollino et al. 2020, Cabras and Medina 2019, Cabras et al. 2021, Cabras et al. 2020, Schultze 1925, Yap 2008. There are already more than 230 species of *Metapocyrtus* documented in the Philippines (Bollino et al. 2020, Cabras et al. 2021, Cabras et al. 2018,Schultze 1925, Yap 2008) with new species continually being discovered (Yoshitake 2011, Yoshitake 2017, Cabras et al. 2018, Cabras and Medina 2019, Cabras et al. 2021, Cabras et al. 2020, Bollino et al. 2020). In this paper, the new species is described with brief notes on its ecology. The restricted distribution, and incessant habitat loss, combined with the high demand amongst hobbyists and collectors makes this group Vulnerable (Department of Environmental and Natural Resources 2019) and in need of immediate conservation measures.

## Materials and methods

Prior to the field expedition, Gratuitous Permit was obtained from the Department of Environment and Natural Resources Region XI. The specimens deposited in the University of Mindanao Coleoptera Research Center were collected through sheet beating and handpicking. Vials containing 95% ethyl alcohol were used for initial storage immediately after collection. The specimens were examined under a Luxeo 4D and Nikon SMZ745T stereomicroscope for their description. The illustrations, as well as the treatment of the genitals, were identical to those described by Yoshitake (2011). Due to the little or almost no use of the female genitalia in identifying and characterising the different species of Pachyrhynchini (Bollino et al. 2020), the said anatomical parts are no longer illustrated. The specimens were then cleaned and air dried before high resolution photographs of the habitus and aedeagus were taken using a Canon EOS 800D digital camera with a Canon MP-E 65 mm macrolens, then stacked and processed using licensed Helicon Focus Pro 7.6.6 and Photoshop CS6. Then, the specimens were mounted for deposition. Label data are cited verbatim. In the text, we used the following symbols and abbreviations:

- / = different lines
- // = different labels
- â: = arithmetic mean
- LB = length of the body in dorsal view, from the apical margin of the pronotum to the apices of the elytra
- LE = length of the elytra in dorsal view, from the level of the basal margins to the apices of the elytra
- LP = length of the pronotum, from the base to apex along the mid-line
- LR = length of the rostrum
- WE = maximum width across the elytra
- WP = maximum width across the pronotum
- WR = maximum width across the rostrum

All measurements are in millimetres.

Codes of the collections:

- RJTV – Private Collection of Reagan Joseph T. Villanueva, Davao City, Philippines.
- UMCRC – University of Mindanao Coleoptera Research Center, Davao City, Philippines.

## Taxon treatments

### Metapocyrtus dagtum

Cabras, Torrejos & Medina sp. n.

FD74BB62-E0F0-5B31-AAFF-E53CFB93862A

15B8E17A-6CDD-4150-858D-C49013CBA40D

#### Materials

- **Type status:** Holotype. **Occurrence:** recordedBy: Local Collector; individualCount: 1; sex: male; lifeStage: adult; **Taxon:** scientificName: Metapocyrtusdagtum; kingdom: Animalia; phylum: Arthropoda; class: Insecta; order: Coleoptera; family: Curculionidae; genus: Metapocyrtus; specificEpithet: dagtum; **Location:** continent: Asia; islandGroup: Mindanao; country: Philippines; countryCode: PHL; stateProvince: Davao de Oro; municipality: Maragusan; locality: Mt. Candalaga; locationRemarks: Type on red white card: Deposited in UMCRC; **Identification:** identifiedBy: AA Cabras; **Event:** samplingProtocol: handpicking; year: 2019; month: June
- **Type status:** Paratype. **Occurrence:** recordedBy: RJTV; individualCount: 8; sex: male; lifeStage: adult; **Taxon:** scientificName: Metapocyrtusdagtum; kingdom: Animalia; phylum: Arthropoda; class: Insecta; order: Coleoptera; family: Curculionidae; genus: Metapocyrtus; specificEpithet: dagtum; **Location:** continent: Asia; islandGroup: Mindanao; country: Philippines; countryCode: PHL; stateProvince: Davao de Oro; municipality: New Bataan; locality: Shadol; locationRemarks: Type on red white card: Deposited in UMCRC; **Identification:** identifiedBy: AA Cabras; **Event:** samplingProtocol: handpicking; year: 2019; month: June
- **Type status:** Paratype. **Occurrence:** recordedBy: RJTV; individualCount: 5; sex: female; lifeStage: adult; **Taxon:** scientificName: Metapocyrtusdagtum; kingdom: Animalia; phylum: Arthropoda; class: Insecta; order: Coleoptera; family: Curculionidae; genus: Metapocyrtus; specificEpithet: dagtum; **Location:** continent: Asia; islandGroup: Mindanao; country: Philippines; countryCode: PHL; stateProvince: Davao de Oro; municipality: New Bataan; locality: Shadol; locationRemarks: Type on red white card: Deposited in UMCRC; **Identification:** identifiedBy: AA Cabras; **Event:** samplingProtocol: handpicking; year: 2019; month: June
- **Type status:** Paratype. **Occurrence:** recordedBy: Local Collector; individualCount: 8; sex: male; lifeStage: adult; **Taxon:** scientificName: Metapocyrtusdagtum; kingdom: Animalia; phylum: Arthropoda; class: Insecta; order: Coleoptera; family: Curculionidae; genus: Metapocyrtus; specificEpithet: dagtum; **Location:** continent: Asia; islandGroup: Mindanao; country: Philippines; countryCode: PHL; stateProvince: Davao de Oro; municipality: Maragusan; locality: Langgawisan; locationRemarks: Type on red white card: Deposited in UMCRC; **Identification:** identifiedBy: AA Cabras; **Event:** samplingProtocol: handpicking; year: 2019; month: October
- **Type status:** Paratype. **Occurrence:** recordedBy: Local Collector; individualCount: 5; sex: female; lifeStage: adult; **Taxon:** scientificName: Metapocyrtusdagtum; kingdom: Animalia; phylum: Arthropoda; class: Insecta; order: Coleoptera; family: Curculionidae; genus: Metapocyrtus; specificEpithet: dagtum; **Location:** continent: Asia; islandGroup: Mindanao; country: Philippines; countryCode: PHL; stateProvince: Davao de Oro; municipality: Maragusan; locality: Langgawisan; locationRemarks: Type on red white card: Deposited in UMCRC; **Identification:** identifiedBy: AA Cabras; **Event:** samplingProtocol: handpicking; year: 2019; month: October
- **Type status:** Paratype. **Occurrence:** recordedBy: Local Collector; individualCount: 1; sex: male; lifeStage: adult; **Taxon:** scientificName: Metapocyrtusdagtum; kingdom: Animalia; phylum: Arthropoda; class: Insecta; order: Coleoptera; family: Curculionidae; genus: Metapocyrtus; specificEpithet: dagtum; **Location:** continent: Asia; islandGroup: Mindanao; country: Philippines; countryCode: PHL; stateProvince: Davao de Oro; municipality: Maragusan; locality: Mt. Candalaga; locationRemarks: Type on red white card: Deposited in UMCRC; **Identification:** identifiedBy: AA Cabras; **Event:** samplingProtocol: handpicking; year: 2019; month: September
- **Type status:** Paratype. **Occurrence:** recordedBy: RJTV; individualCount: 2; sex: male; lifeStage: adult; **Taxon:** scientificName: Metapocyrtusdagtum; kingdom: Animalia; phylum: Arthropoda; class: Insecta; order: Coleoptera; family: Curculionidae; genus: Metapocyrtus; specificEpithet: dagtum; **Location:** continent: Asia; islandGroup: Mindanao; country: Philippines; countryCode: PHL; stateProvince: Davao de Oro; municipality: New Bataan-Maragusan Highway Boundary; locationRemarks: Type on red white card: Deposited in UMCRC; **Identification:** identifiedBy: AA Cabras; **Event:** samplingProtocol: handpicking; year: 2016; month: June
- **Type status:** Paratype. **Occurrence:** recordedBy: Local Collector; individualCount: 1; sex: male; lifeStage: adult; **Taxon:** scientificName: Metapocyrtusdagtum; kingdom: Animalia; phylum: Arthropoda; class: Insecta; order: Coleoptera; family: Curculionidae; genus: Metapocyrtus; specificEpithet: dagtum; **Location:** continent: Asia; islandGroup: Mindanao; country: Philippines; countryCode: PHL; stateProvince: Davao de Oro; municipality: Maragusan; locality: Mahayahay; locationRemarks: Type on red white card: Deposited in UMCRC; **Identification:** identifiedBy: AA Cabras; **Event:** samplingProtocol: handpicking; year: 2018; month: June
- **Type status:** Paratype. **Occurrence:** recordedBy: Local Collector; individualCount: 1; sex: female; lifeStage: adult; **Taxon:** scientificName: Metapocyrtusdagtum; kingdom: Animalia; phylum: Arthropoda; class: Insecta; order: Coleoptera; family: Curculionidae; genus: Metapocyrtus; specificEpithet: dagtum; **Location:** continent: Asia; islandGroup: Mindanao; country: Philippines; countryCode: PHL; stateProvince: Davao de Oro; municipality: New Bataan-Maragusan Highway Boundary; locationRemarks: Type on red white card: Deposited in UMCRC; **Identification:** identifiedBy: AA Cabras; **Event:** samplingProtocol: handpicking; year: 2018; month: June

#### Description

**Male**

Dimensions (in mm): N=20. LB: 9.3–11.6 (holotype 9.3), LR: 1.9–2.7 (1.9), WR: 1.2–1.8 (1.2), LP: 3.0–3.9 (3.0), WP: 3.2–4.5 (3.2), LE: 7.2–7.7 (7.2), WE: 4.3–5.1 (4.3).

**Figure 1.**
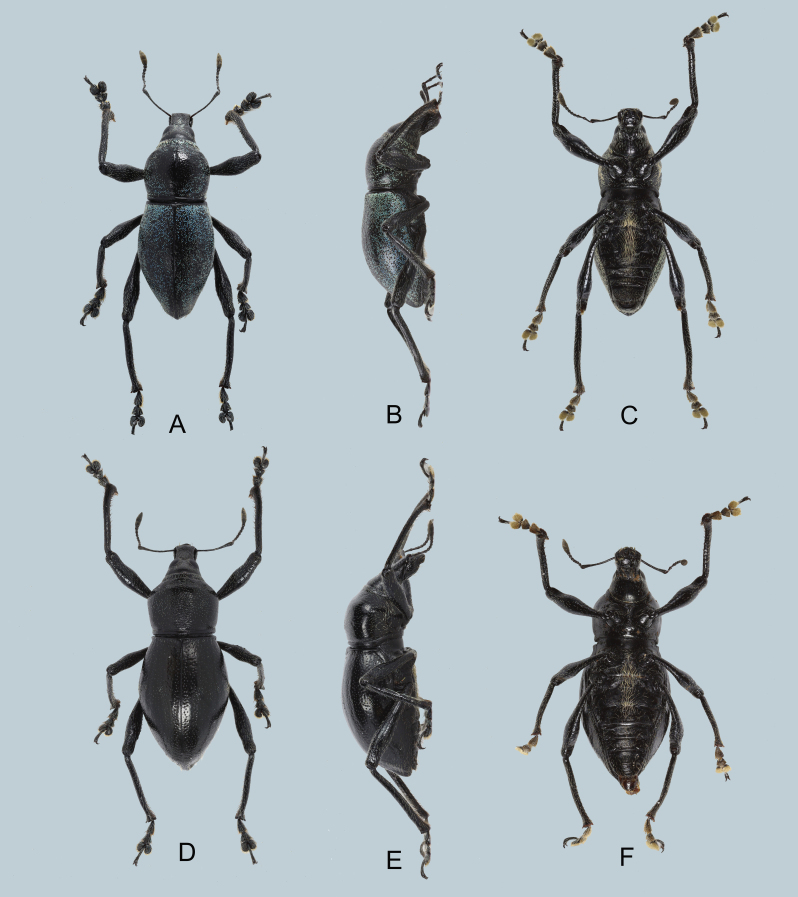
*Metapocyrtusdagtum* sp. nov. **A–C** Male, holotype: **A** dorsal view, **B** lateral view, **C** ventral view **D–F** Female, paratype, **A** dorsal view, **E** lateral view, **F** ventral view.

Habitus as shown in Fig. 1A–C.

Integument black. Body surface, rostrum, head and underside with weak lustre. Body subglabrous. Head subglabrous with pale-blue, lanceolate scales and adpressed white piliform scales on forehead and coloured setae on sides; forehead weakly depressed, rugose especially towards eyes, and with faint longitudinal groove not reaching vertex.

Rostrum moderately rugose, longer than wide (LR/WR: 1.58), bearing sparse and adpressed piliform scales on dorsum and long white piliform scales on lateral surface below antennal scrobe; anterolateral sides also covered with long white piliform scales; transverse basal groove distinct; longitudinal groove barely present, but replaced with weak depression; apical third of dorsum finely punctured; dorsal surface nearly flat. Eyes medium-sized and feebly convex. Antennal scape slightly longer than the funicle (scape/funicle: 2.0/1.6). Scape moderately covered with fine, white adpressed hairs and funicle with yellowish suberect hairs. Funicular antennomeres I and II nearly the same length, twice as long as wide; funicular antennomeres III–VII nearly as long as wide; club subellipsoidal, nearly three times longer than wide.

Prothorax subglobular, wider than long (LP/WP: 0.94), subglabrous, weakly punctured, weakly rugose near anterior and posterior margins, widest at mid-length, weakly convex, highest point at mid-length, lateral margins convexly rounded and posterior margin truncate. Pronotum covered with pale blue, pale yellow and bluish-green lanceolate scales and adpressed, coloured piliform scales, except along midline which is nearly bare; anterior margin with yellow-ochre and pale blue narrowly ovate scales, and lateral sides above coxae covered with yellow-ochre narrowly ovate scales.

Elytra narrowly subovate, moderately tapered towards apex (LE/WE: 1.67), slightly wider and nearly twice longer than prothorax (WE/WP: 1.34, LE/LP: 2.40), subglabrous and weakly convex; surface black, irregularly covered with pale blue, turquoise and dull yellow, round to ovate scales and coloured adpressed piliform scales; apex with very sparse, coloured, fine setae.

Legs with moderately clavate femora. Femora fairly covered with white adpressed piliform scales. Tibiae fairly covered with adpressed, white piliform scales; piliform scales longer along inner margin; weakly serrate along inner edge. Fore- and mid-tibiae bearing mucro at apex. Tarsomeres pubescent all throughout. Coxae pubescent all throughout with white piliform scales. Mesoventrite covered with white adpressed piliform scales. Metaventrite with concentration of long golden-yellow adpressed piliform scales on discs, and turquoise, light-blue and dull yellow, round scales at distal ends. Ventrite I depressed on disc, with concentration of long golden-yellow adpressed piliform scales. Ventrites II–V with concentration of golden yellow hair-like scales, but much shorter. Ventrite V flattened, apex with fine punctures and suberect white piliform scales.

**Figure 2.**
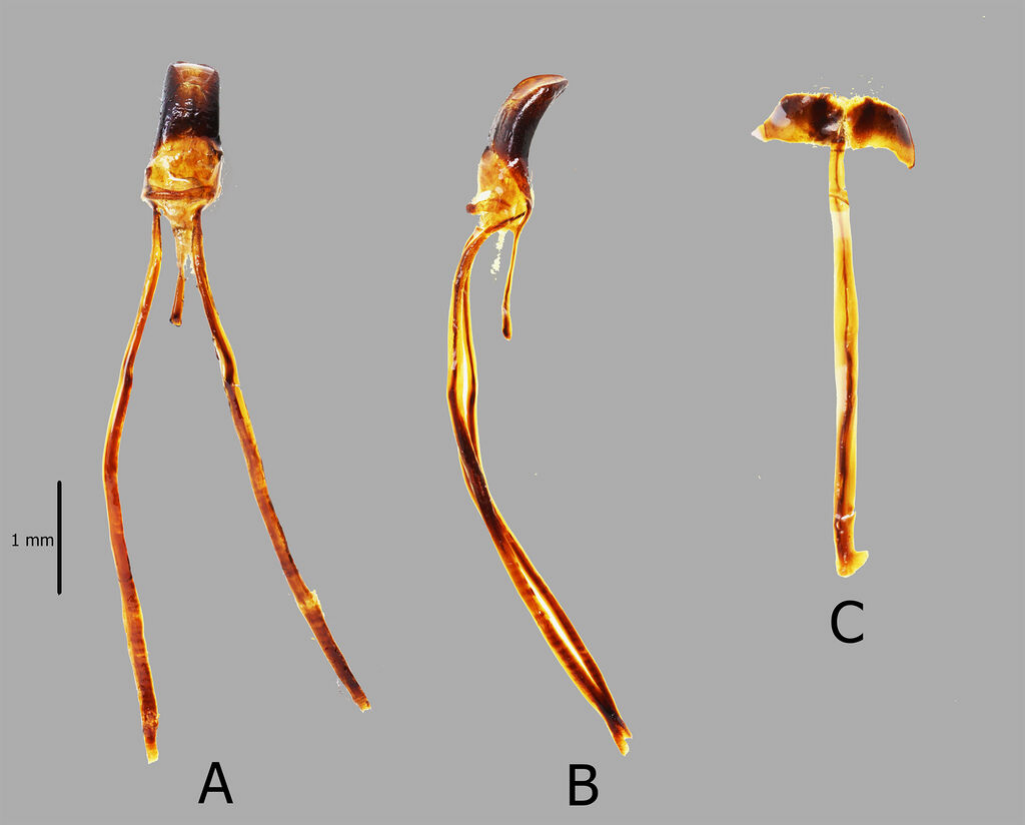
Male genitalia of *Metapocyrtusdagtum* sp. nov. **A** aedeagus in dorsal view **B** aedeagus in lateral view **C** sternite IX in dorsal view.

**Male genitalia** as shown in Fig. 2 (A–C). Aedeagal body short and stout, apex rounded, and apodemes nearly four times longer than aedeagal body.

**Female**

Dimensions (in mm): N:11. LB: 9.6–11.1 (â: 10.3), LR: 2.2–2.3 (2.2), WR: 1.2–1.5 (1.3 mm), LP: 2.4–3.5 (2.8 mm), WP: 3.3–4.4 (3.7 mm), LE: 7.2–8.5 (7.6 mm), WE: 5.0–5.7 (5.2 mm).

Habitus as shown in Fig. 1D-F.

Females differ from males in the following: a) rostrum slightly shorter in females; b) pronotum slightly shorter than in male (LP/WP: 0.67–0.79: female, 0.94: male); c) pronotum subquadrate with widest point right after anterior margin and truncate towards posterior margin; d) pronotum rugose at middle of disc; e) absence of lanceolate and ovate scales (which are part of the variation of elytral scales of this species); f) elytra subovate (LE/WE: 1.44–1.49), wider than in male (WE/WP: 1.29–1.51, LE/LP 2.42–3.0), widest before mid-legth and less constricted towards apex, and g) elytra with minute pubescence. Other than those features, females are similar to males.

##### Variation

**Figure 3.**
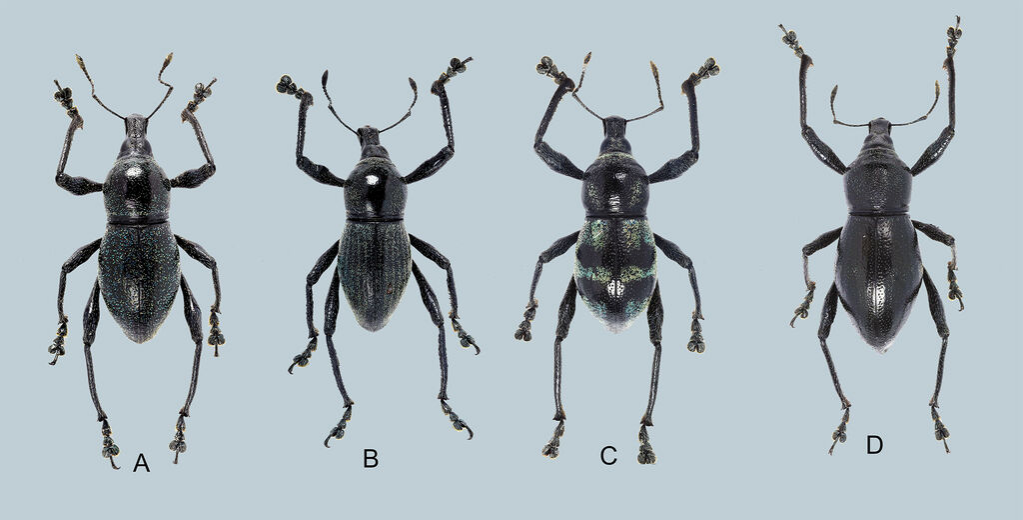
Variability of elytral markings of *Metapocyrtusdagtum* sp. nov.: **A–C**, male habitus, D, female habitus.

*Metapocyrtusdagtum* sp. nov. displays a striking variation in its elytral pattern, as well as the shape of its scale markings (Fig. 3). While the holotype is observed to have pale blue, turquoise and dull yellow round to ovate scales on the elytra, prominently concentrated on its basal and outer margins, other specimens have sparser scales, while others are almost bare and other specimens with scales concentrated on the elytral striae. The scales also vary from round to ovate to piliform. Around 10% of the specimens also appear to have three transverse scale bands on the elytra consisting of a wide basal transverse band which extends at least 0.5 mm from the elytral suture to the lateral margins, a narrow transverse band on the middle and a subtriangular band along the elytral apex, all confluent towarss the lateral margin.

#### Diagnosis

*Metapocyrtusdagtum* sp. nov. is readily distinguished from its congeners by the unique shape of the pronotum and body, as well as scaly markings. The new species resembles Metapocyrtus (Metapocyrtus) lindabonus Schultze, 1922 by the aedeagus and both having a glossy black body with slender and subovate elytra, and a subglobular pronotum which is truncated at the base. *Metapocyrtusdagtum* sp. nov. can be easily distinguished from M. (M.) lindabonus by its longer and slender rostrum, absence of two scaly spots on both sides of the disc, strongly truncate pronotum, tapered elytral apex, and the uniform presence of piliform or round scales on the elytra.

#### Etymology

The specific epithet is from the Cebuano word “dagtum” which means pitch black, referring to the colour of the integument of the species.

#### Distribution

**Figure 4.**
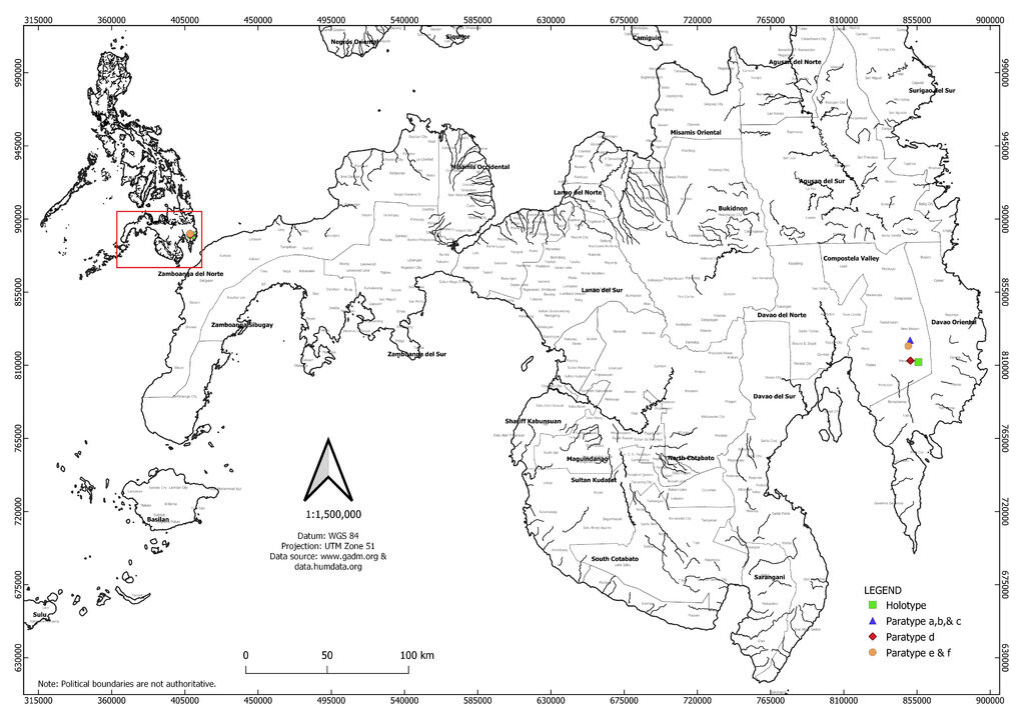
Distribution map of *Metapocyrtusdagtum* sp.nov.

The new species is known only from the type locality in Davao de Oro, Mindanao, Philippines at present. The type localities of *Metapocyrtusdagtum* sp. nov. are in Maragusan and New Bataan which are located in the south-eastern portion of Davao de Oro (Fig. 4).

#### Biology

**Figure 5.**
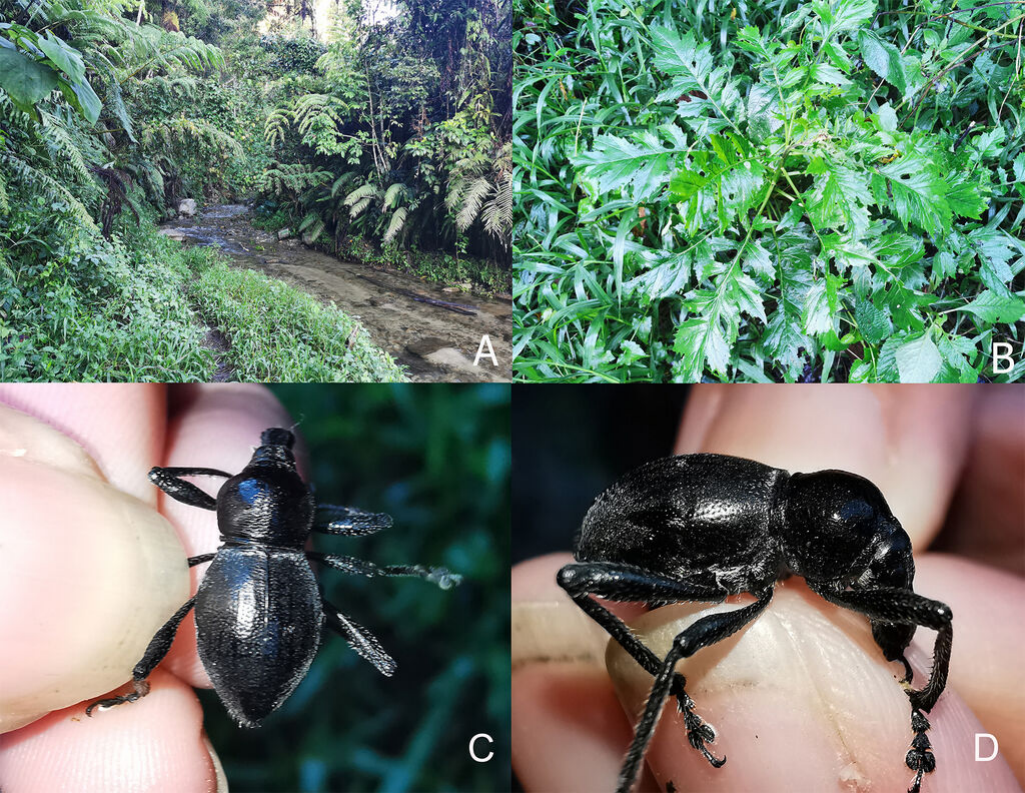
Habitat and habitus of *Metapocyrtusdagtum* sp. nov. **A** Habitat in Langgawisan, Maragusan, Davao de Oro, Mindanao **B** *Erechtitesvalerianifolius* (Link ex Spreng.) DC., a possible food plant of *Metapocyrtusdagtum* sp. nov. **C–D** *Metapocyrtusdagtum* sp. nov. in the field.

The new species was collected in the partially-shaded creek whose nearby ecotypes include agricultural and mixed secondary forests. The shallow creek’s vegetation includes various shrubs and ferns, such as *Cyathea* sp. (Cyatheaceae), *Angiopterisevecta* (Marattiaceae), and *Diplaziumesculentum* (Woodsiaceae), amongst others. The new species was, however, collected on the plant *Erechtitesvalerianifolius* (Asteraceae), along the banks of the stream (Fig. 5).

## Supplementary Material

XML Treatment for Metapocyrtus dagtum
